# Hierarchical Ridge Regression for Incorporating Prior Information in Genomic Studies

**DOI:** 10.6339/21-jds1030

**Published:** 2021-12-13

**Authors:** Eric S. Kawaguchi, Sisi Li, Garrett M. Weaver, Juan Pablo Lewinger

**Affiliations:** 1 Department of Population and Public Health Sciences, Keck School of Medicine, University of Southern California, Los Angeles, California, USA

**Keywords:** high-dimensional regression, meta-features, penalization, prediction, regularization

## Abstract

There is a great deal of prior knowledge about gene function and regulation in the form of annotations or prior results that, if directly integrated into individual prognostic or diagnostic studies, could improve predictive performance. For example, in a study to develop a predictive model for cancer survival based on gene expression, effect sizes from previous studies or the grouping of genes based on pathways constitute such prior knowledge. However, this external information is typically only used post-analysis to aid in the interpretation of any findings. We propose a new hierarchical two-level ridge regression model that can integrate external information in the form of “meta features” to predict an outcome. We show that the model can be fit efficiently using cyclic coordinate descent by recasting the problem as a single-level regression model. In a simulation-based evaluation we show that the proposed method outperforms standard ridge regression and competing methods that integrate prior information, in terms of prediction performance when the meta features are informative on the mean of the features, and that there is no loss in performance when the meta features are uninformative. We demonstrate our approach with applications to the prediction of chronological age based on methylation features and breast cancer mortality based on gene expression features.

## 1 Introduction

In genomic studies, there is often a great deal of prior knowledge about the genomic features that are being modeled. These “meta features” (or features-of-features) may be comprised of gene annotations (e.g., an indicator to denote whether a gene belongs to a particular pathway), natural groupings of the genomic features (e.g., methylation probes mapping to genes), or information from previous studies (e.g., scores or effect estimates of a SNP on the outcome) that the researcher considers relevant to the outcome of interest. For example, the Molecular Taxonomy of Breast Cancer International Consortium (METABRIC) study includes cDNA microarray profiling of close to two thousand breast cancer patients and patients’ survival information within the study follow-up (Curtis et al., 2012). In this example, which we later use to illustrate our approach, we are interested in predicting patient mortality based on their gene expression profiles. As potentially informative meta features we consider the attractor metagenes identified by Cheng et al. (2013). These are groups of genes that capture molecular events known to be associated with clinical outcomes in many cancers. We expect improved prediction performance when incorporating these metagenes into the model building process.

Genomic data are often high-dimensional i.e., has more features per observation than observations in the study. But classical regression methods such as linear and logistic regression breakdown in high-dimensional settings. High-dimensional regression methods require regularization, a technique that modifies the loss function by adding a penalty term that shrinks the regression coefficients toward zero. Among the best known examples of regularized/penalized regression are ridge regression (Hoerl and Kennard, 1976), LASSO (Tibshirani, 1996), and elastic net (Zou and Hastie, 2005), though many other approaches have been developed to encourage additional structure or desirable properties of the regression estimates (e.g., Fan and Li, 2001; Yuan and Lin, 2006; Zou, 2006; Zhang, 2010; Dai et al., 2018). The amount of shrinkage induced by the penalty dictates the balance between model complexity (bias) and model stability (variance). It is controlled by a penalty parameter that requires tuning, which is typically accomplished via cross-validation.

While most regularization methods penalize all regression coefficients equally, feature-specific weighting can be performed to allow for differential shrinkage. In particular, several approaches have been recently proposed to improve the prediction performance of regularized regression models through the integration of prior information. Using the LASSO (Tibshirani, 1996) framework, Bergersen et al. (2011) incorporates relevant meta features by developing feature-specific penalties. This modification provided more stable model selection and improved prediction over the standard LASSO. Similarly, Van De Wiel et al. (2016) proposed an adaptive group-regularized version of ridge (Hoerl and Kennard, 1976) regression which derives empirical Bayes estimates for group-specific penalties by utilizing meta features such as gene annotations or external p-values. Recently, Tay et al. (2021) proposed the feature-weighted elastic net that uses meta features to adapt the feature-specific penalties for elastic net (Zou and Hastie, 2005) regularization and Zeng et al. (2020) proposed an alternative approach that models the magnitude of the subject-specific tuning parameters as a log-linear function of the meta features.

Some of these approaches fix the weights in advance (e.g. Bergersen et al., 2011), which requires unavailable knowledge about the *relative* importance of the features. Others, adaptively (re)-estimate these weights (see e.g., Van De Wiel et al., 2016; Tay et al., 2021; Zeng et al., 2020), but this requires tuning a potentially large number of parameters, which in turn limits the number of meta features that can be integrated at any given time. In addition, by modifying the penalties, these methods assume that the meta features are explaining variations in the features. Instead of using the meta features to determine weights, we propose a hierarchical *ℓ*_2_-regularized (two-level ridge regression) model that jointly models the subject-level features and meta features, which enables the integration of any type and number of meta features. At the first level, the outcome is regressed on the subject-level features, as in standard regularization methods. Rather than assuming the meta features affect the variance of the subject-level features, the second level models the effect of the meta features on the mean of the subject-level features. *L*_2_-regularization is applied to the subject-level features and the meta features as both sets (features and meta features) have the potential to be highly correlated and high dimensional. We show that the two-level ridge regression model can be rewritten as a single ridge regression with a modified design matrix and parameter vector, which allows us to use efficient optimization techniques to estimate the model parameters. We also derive closed-form solutions under specific scenarios that sheds light on how the external information impacts estimation of the first-level regression coefficients.

The rest of the paper is organized as follows. The two-level ridge regression model is described in Section 2. In Section 3, we provide a simulation study that compares our proposed method to competing methods. Real data applications for predicting chronological age and breast cancer mortality are given in Section 4. Discussions of our findings and parting comments are provided in Section 5. The two-level ridge regression model is implemented in the R package *xrnet* (Weaver and Lewinger, 2019, 2021), which can be found at https://CRAN.R-project.org/package=xrnet.

## 2 Methods

### 2.1 Setup

Consider the linear regression model

(1) y=Xβ+ϵ,

where $y\in {\mathbb{R}}^{n}$ is a vector of quantitative measurements collected on *n* subjects, $X=\left(x_{1}^{T},\ldots ,x_{n}^{T}\right)$ is an *n* × *p* matrix of genomic features (e.g., expression levels, genotypes, methylation probes), ***β*** = (*β*_1_, …, *β*_*p*_)^*T*^ is the vector of regression coefficients, and ***ϵ*** ~ 𝒩_*p*_(0, *σ*^2^*I*_*p*_) for some *σ*^2^ > 0. We assume, for notational convenience, that the observations are standardized with sample mean 0 (which removes the intercept term) and sample variance 1. The genomic features are assumed to be high-dimensional, i.e., the number of features *p* exceeds the sample size *n*.

We also assume that there is a set of *q* meta features (e.g., gene annotations, natural groupings, information from previous studies) collected for each of the *p* features that can be represented as a *p* × *q* matrix *Z*. The number of meta-features can be larger than *p* and/or *n*. Our goal is to improve the prediction performance by integrating the meta features into the following modeling framework.

### 2.2 The Model

In a high-dimensional setting, unique ordinary least squares estimates for model 1 do not exist. Essentially, the linear regression model with more features than observations is too complex for the amount of data available. As mentioned in the introduction, regularization methods (see e.g., Hoerl and Kennard, 1976; Tibshirani, 1996; Fan and Li, 2001; Zou and Hastie, 2005; Zou, 2006; Zhang, 2010; Dai et al., 2018) address this issue by balancing model complexity/parsimony and goodness of fit. Initially developed for handling multicollineairity, ridge regression (Hoerl and Kennard, 1976) is an effective approach for analyzing high-dimensional data. Ridge regression is the solution to an optimization problem with a modified objective function that adds an *ℓ*_2_-penalty to the standard squared loss function:

(2) $${\hat{\beta }}^{\text{ridge}}=\text{arg }\underset{\beta }{\text{min}}\frac{1}{2}\|\text{y}-X\beta {\|}_{2}^{2}+\frac{\lambda }{2}\|\beta {\|}_{2}^{2}\text{,}$$

where $\|\beta {\|}_{2}^{2}=\sum_{j=1}^{p}\beta_{j}^{2}$ and *λ* ⩾ 0. The *ℓ*_2_ penalty encourages shrinkage of the coefficient estimates toward zero and the degree of shrinkage is controlled by the choice of the tuning parameter *λ* (see Section 2.4). A common approach to tune *λ* is to select the value that minimizes some criterion (e.g., mean squared error) from a grid of possible values of *λ* using *k*-fold cross validation.

To incorporate meta features into high-dimensional linear regression, we propose a two-level *ℓ*_2_-regularization approach based on minimizing the following objective function

(3) $$\text{arg }\underset{\beta ,\gamma }{\text{min}}\left\{\frac{1}{2}\|y-X\beta {\|}_{2}^{2}+\frac{\lambda_{1}}{2}\|\beta -Z\gamma {\|}_{2}^{2}+\frac{\lambda_{2}}{2}\|\gamma {\|}_{2}^{2}\right\},$$

where *λ*_1_ > 0 and *λ*_2_ > 0 are two tuning parameters. The first term in (3) is the standard least squares loss, the second term is a ridge penalty that shrinks the estimates of ***β*** toward some feature-specific mean ***μ*** = *Z****γ*** (rather than **0**), and the third term is a standard ridge penalty that shrinks the estimates of ***γ***. Note that unlike standard ridge regression, the value of ***μ*** toward which the ***β*** are shrunk is not fixed but modeled as a linear function of the meta features *Z*. This second-level penalty encourages genomic features with similar meta-feature profiles to have more similar coefficient estimates compared to genomic features with dissimilar profiles, effectively “borrowing information” across features. We provide specific examples in Section 2.4. Note also that when ***γ*** = **0**, (3) reduces to (2), and thus the standard ridge regression is a particular submodel of our hierarchical formulation. Furthermore, the second term can be viewed as a least squares regression of ***β*** on *Z*. In this case, ***β*** takes the role of the “outcome”. A Bayesian motivation behind this hierarchical formulation is provided in the Online Supplementary Materials. Under the Bayesian framework, it is clear that (3) assumes the meta features affect the mean of the subject-level features. This is in contrast to other approaches that integrate meta features by creating feature-specific penalties which, consequently, assumes that the meta features impact the variance of the subject-level features. Shrinkage of both the subject-level features (to the feature-specific mean ***μ***) and meta features (to **0**) is controlled by *λ*_1_ and *λ*_2_, respectively. Similar to the standard ridge regression, one can use *k*-fold cross validation to select the optimal pair of values for *λ*_1_ and *λ*_2_ over a two-dimensional grid.

While equation (3) posits a natural hierarchical structure to the model, the objective function can be simplified to a single linear regression model using the following variable substitution, ***ϕ*** = ***β*** − *Z****γ***. By jointly minimizing over (***ϕ***, ***γ***), (3) can be rewritten as

(4) $$\text{arg }\underset{\phi ,\gamma }{\text{min}}\left\{\frac{1}{2}\|y-X(\phi +Z\gamma ){\|}_{2}^{2}+\frac{\lambda_{1}}{2}\|\phi {\|}_{2}^{2}+\frac{\lambda_{2}}{2}\|\gamma {\|}_{2}^{2}\right\}.$$

The formulation in (4) can be extended to include penalties other than ridge. In fact, commonly-used penalties such as the LASSO or elastic-net could be used for regularization on either (or both) the subject-level or meta feature coefficients. We focus on *ℓ*_2_ regularization on both levels due to its ability to handle highly-correlated features (Zou and Hastie, 2005) and its generally good performance in prediction problems.

### 2.3 Model Fitting

Since (4) is jointly convex in (***ϕ***, ***γ***) it can be minimized using standard convex optimization methods. In particular, being also separable, cyclic coordinate descent can be used to efficiently optimize it with guaranteed convergence to a global minimum (Tseng, 2001). Before outlining the algorithm, we further simplify the notation by letting $\tilde{X}=[X,XZ]$ and ***θ*** = (***ϕ***, ***γ***)^*T*^. We can then re-express (4) as

(5) $$\frac{1}{2}\|y-X(\phi +Z\gamma ){\|}_{2}^{2}+\frac{\lambda_{1}}{2}\|\phi {\|}_{2}^{2}+\frac{\lambda_{2}}{2}\|\gamma {\|}_{2}^{2}\;=\frac{1}{2}\|y-\tilde{X}\theta {\|}_{2}^{2}+\frac{1}{2}\theta^{T}\Lambda \theta ,$$

where Λ = *diag*(Λ_1_, Λ_2_), Λ_1_ = *diag*(*λ*_1_, …, *λ*_1_) and Λ_2_ = *diag*(*λ*_2_, …, *λ*_2_).

In summary, our two-level ridge regression model can be reformulated as a single-level ridge regression, where the first *p* variables, *X*, have a specific penalty parameter, *λ*_1_, and the last *q* variables, *XZ*, have a specific penalty parameter *λ*_2_. It may seem that (5) provides a framework for differential *ℓ*_2_ regularization of multi-omic data (e.g., Gross and Tibshirani, 2015; Chai et al., 2017; Liu et al., 2018). While multi-omic data refers to a collection of multiple subject-level measurements, our hierarchical formulation assumes that we have one set of measurements at the subject level (*X*) and one set of meta features at the feature level (*Z*). Since the rows of the *XZ* matrix are linear combinations of the original features given by the columns of *Z*, it is never full rank even when *p* + *q* < *n*. Shrinkage is necessary to produce unique estimates, even in the low dimensional case. Furthermore, (5) admits the following closed-form solution,

$$\hat{\theta }={\left({\tilde{X}}^{T}\tilde{X}+\Lambda \right)}^{-1}{\tilde{X}}^{T}y.$$

which can be computed using numerical linear algebra. In practice, however, we propose to employ cyclic coordinate descent due to its efficiency in generating entire solution paths across a grid of tuning parameters through the use of warm starts (Friedman et al., 2010) and for its generalizability to other outcome types (see Section 2.5). We outline the cyclic coordinate descent algorithm in the Online Supplementary Materials.

The formulation of (5) allows it to be solved using currently-available software (e.g., glmnet) for fixed values of (*λ*_1_, *λ*_2_). However, an important distinction is that we allow ***ϕ*** to be penalized differently than ***γ***. We demonstrate this in our simulation study. The cyclic coordinate descent algorithm simultaneously estimates ***ϕ*** and ***γ***. Estimates of ***β*** can be obtained by the back transformation $\hat{\beta }=\hat{\phi }+Z\hat{\gamma }$. Our implementation estimates the model parameters for a two-dimensional grid of penalty tuning parameters (*λ*_1_, *λ*_2_) and performs joint parameter tuning of *λ*_1_ and *λ*_2_ using cross validation.

### 2.4 Behavior of the Two-Level Ridge Regression Model

When the matrix *X* is of full column rank (i.e. well-conditioned low-dimensional case), we can investigate the relationship between both the ridge and ordinary least squares solutions. Under an orthonormal design matrix (i.e. *X*^*T*^ *X* = *I*_*p*_) the ridge estimator has the explicit solution:

(6) $${\hat{\beta }}^{\text{ridge}}=\frac{1}{1+\lambda }{\hat{\beta }}^{ols}\text{,}$$

where ${\hat{\beta }}^{ols}$ are the least squares estimates. Therefore, one can see that for *λ* → 0, ${\hat{\beta }}^{\text{ridge}}\to {\hat{\beta }}^{ols}$ and for *λ* → ∞, ${\hat{\beta }}^{\text{ridge}}\to 0$.

Similar to the closed form solution in (6), under the single-level formulation (5) we can derive closed-form solutions for the parameters estimates, under certain assumptions, that reveals how the external information in *Z* impacts estimation of the coefficients ***β***. While we let *X* denote generic genomic features, for concreteness, we present the following examples in terms of gene expression levels.

#### 2.4.1 Case 1: Disjoint Groups (E.g., Gene Expression for Genes in Non-Overlapping Pathways)

Let *X* be an *n* × 4 orthogonal design matrix (i.e., *X*^*T*^ *X* = *I*_4_) of gene expression levels. Suppose that the first two genes belong to one specific pathway and the last two genes belong to another pathway, disjoint from the first. Then *Z* can be expressed as a 4 × 2 matrix of binary indicators:

$$Z=\left[\begin{array}{ll} 1 & 0\\ 1 & 0\\ 0 & 1\\ 0 & 1 \end{array}\right]$$

By solving (5), one can show that the estimates for ***β*** are

$$\left(\begin{array}{l} {\hat{\beta }}_{1}\\ {\hat{\beta }}_{2}\\ {\hat{\beta }}_{3}\\ {\hat{\beta }}_{4} \end{array}\right)=\left(\begin{array}{l} {\hat{\beta }}_{1}^{\text{ridge}}+\lambda^{*}\left({\hat{\beta }}_{1}^{\text{ridge}}+{\hat{\beta }}_{2}^{\text{ridge}}\right)\\ {\hat{\beta }}_{2}^{\text{ridge}}+\lambda^{*}\left({\hat{\beta }}_{1}^{\text{ridge}}+{\hat{\beta }}_{2}^{\text{ridge}}\right)\\ {\hat{\beta }}_{3}^{\text{ridge}}+\lambda^{*}\left({\hat{\beta }}_{3}^{\text{ridge}}+{\hat{\beta }}_{4}^{\text{ridge}}\right)\\ {\hat{\beta }}_{4}^{\text{ridge}}+\lambda^{*}\left({\hat{\beta }}_{3}^{\text{ridge}}+{\hat{\beta }}_{4}^{\text{ridge}}\right) \end{array}\right),$$

where $\lambda^{*}=\frac{\lambda_{1}^{2}}{2\lambda_{1}+\lambda_{1}\lambda_{2}+\lambda_{2}}$. Thus we see that the subject-level estimates are equal to their standard ridge estimator plus a weighted sum of the estimates in the same pathway.

#### 2.4.2 Case 2: Genes in Overlapping Pathways

Our previous example assumed that genes belong to two disjoint pathways, which lends itself to a simple interpretation of the estimators. We assume now that *X* is a *n* × 3 orthogonal design matrix of gene expression levels and let

$$Z=\left[\begin{array}{ll} 1 & 0\\ 1 & 1\\ 0 & 1 \end{array}\right].$$

Unlike the previous example, the second gene belongs now to both pathways. The two-level ridge estimates for this particular scenario are

$$\left(\begin{array}{c} {\hat{\beta }}_{1}\\ {\hat{\beta }}_{2}\\ {\hat{\beta }}_{3} \end{array}\right)=\left(\begin{array}{c} {\hat{\beta }}_{1}^{\text{ridge}}+\lambda^{*}\left(2{\hat{\beta }}_{1}^{\text{ridge}}+{\hat{\beta }}_{2}^{\text{ridge}}-{\hat{\beta }}_{3}^{\text{ridge}}\right)\\ {\hat{\beta }}_{2}^{\text{ridge}}+\lambda^{*}\left({\hat{\beta }}_{1}^{\text{ridge}}+2{\hat{\beta }}_{2}^{\text{ridge}}+{\hat{\beta }}_{3}^{\text{ridge}}\right)\\ {\hat{\beta }}_{3}^{\text{ridge}}+\lambda^{*}\left(-{\hat{\beta }}_{1}^{\text{ridge}}+{\hat{\beta }}_{2}^{\text{ridge}}+2{\hat{\beta }}_{3}^{\text{ridge}}\right) \end{array}\right),$$

where $\lambda^{*}=\frac{\lambda_{1}^{2}}{3\lambda_{1}+\lambda_{1}\lambda_{2}+\lambda_{2}}$. Each ${\hat{\beta }}_{j}$ is now a linear combination (i.e., a weighted sum) of all three ridge estimates.

#### 2.4.3 Case 3: Orthogonal *X* and *Z*

While meta features that define feature groupings are common, meta features of interest can also be quantitative (e.g., test statistics or p-values from previous studies). We now only assume that *Z* is orthogonal to *X*, but can contain quantitative meta features. A general solution in this case is given by:

$$\hat{\beta }=\left(I_{p}+\frac{\lambda_{1}^{2}}{\lambda_{1}\lambda_{2}+\lambda_{1}+\lambda_{2}}ZZ^{T}\right){\hat{\beta }}^{\text{ridge}}$$

The derivation is provided in the Online Supplementary Materials. The first-level coefficient estimates, $\hat{\beta }$, equal their original ridge estimates plus a linear combination of all of the ridge estimates via *ZZ*^*T*^. The matrix *ZZ*^*T*^ can be thought of as a matrix of pairwise similarities between the features, where similarity is measured by the inner product of the pairwise meta-feature profiles. Thus, information is borrowed across all features proportionally to their similarity.

### 2.5 Extension to GLM outcomes

The two-level ridge regression model can be easily extended to models with non-normal outcomes (e.g., binary, categorical, count). Under the generalized linear model framework, we assume that the observations $v_{i}={\left(x_{i}^{T},y_{i}\right)}^{T}$, *i* = 1, …, *n*, are mutually independent and that, conditional on **x**_*i*_, *y*_*i*_ belongs to the exponential family with the following density

(7) $$f_{Y}(y;x,\nu )=\text{exp }\left\{\frac{y\xi -a(\xi )}{b(v)}-c(y,v)\right\},$$

where *ξ* is defined as the canonical parameter, *ν* > 0 is the scale (dispersion) parameter and *a*(*ν*), *b*(*ξ*), and *c*(*y*, *ν*) are known functions whose values depend on the distribution (Dobson and Barnett, 2018; McCullagh, 2019). Furthermore, under the assumption that *a*(·) is twice differentiable, (7) indicates that *E*(*y*_*i*_|**x**_*i*_) = *μ*_*i*_ = *a*′(*ξ*_*i*_) and *var*(*y*_*i*_|**x**_*i*_) = *a*″(*ξ*_*i*_)*b*(*ν*_*i*_). In addition, the canonical parameter *ξ* is connected to **x**_*i*_ through a prespecified link function $h\left(\mu_{i}\right)=x_{i}^{T}\beta$ for some ***β*** = (*β*_1_, …, *β*_*p*_)^*T*^. The likelihood function for ***β*** is defined as

(8) $$L\left(\beta ;v_{i}\right)\propto \overset{n}{\underset{i=1}{\prod }}\text{exp}\left(y_{i}\theta_{i}-a\left(\xi_{i}\right)\right)$$

and the log-likelihood is defined as *l*(***β***) = log *L*(***β***; **v**_*i*_). We estimate the regression coefficients ***β*** by minimizing the negative log-likelihood function. The two-level ridge GLM can now be defined as

(9) $$\text{arg }\underset{\theta }{\text{min}}-l(\theta )+\theta^{T}\Lambda \theta .$$

Since *l*(***θ***) is convex and the ridge penalty is separable, cyclic coordinate descent can again be used to estimate the parameters in the model (see Online Supplementary Materials). We provide an example of the two-level ridge logistic regression in our numerical studies and a real data application on breast cancer mortality is provided.

## 3 Simulation Study

We assess the prediction performance of our proposed two-level ridge estimator to several competing methods: 1) standard ridge regression; 2) “augmented” ridge regression; 3) feature-weighted elastic net (fwelnet); 4) the random forest algorithm. The augmented ridge regression can be viewed as a standard ridge regression (2) with the design matrix $\tilde{X}=[X,XZ]$. While the augmented ridge regression is similar in form to two-level ridge regression (5), the main distinction is that only one tuning parameter is used to shrink both the subject-level and meta-feature effects (***ϕ***, ***γ***). For the random forest algorithm we input the augmented design matrix $\tilde{X}$. For comparison purposes, we fix the elastic net tuning parameter to 0 so that fwelnet will coincide with ridge regularization. Ten-fold cross validation was used to estimate the tuning parameter(s) for the regularization methods. Results are averaged over 500 Monte Carlo replications.

### 3.1 Discrete *Z*

We simulated data loosely based on the breast cancer real data application in Section 4, with gene expression levels as the features and a quantitative outcome. We first consider the case where meta feature matrix *Z* consists of indicator columns corresponding to grouping of genes into (not necessarily disjoint) pathways. Specifically, we generate a binary matrix *Z*_*p*×6_ such that each column has on average 20% nonzero entries where we vary *p* = 400, 1,000, and 2,000. We then set ***γ*** = (0.1, 0.1, 0.1, 0.1, 0.1, 0.1) Conditional on *Z* and ***γ***, we generate the subject-level features by sampling from a multivariate normal distribution $\beta \sim {𝒩}_{p}\left(Z\gamma ,\sigma_{\beta }^{2}I_{p}\right)$. We determined how informative the meta features are for the effect sizes of ***β*** by defining the signal-to-noise ratio (*SNR*_***γ***_) as

$$SNR_{\gamma }=\frac{\gamma^{T}\Sigma_{Z}\gamma }{\sigma_{\beta }^{2}}$$

where Σ_*Z*_ is the empirical covariance matrix of *Z* and solving for $\sigma_{\beta }^{2}$. Finally, we generated the continuous outcome **y**|*X*, $\beta \sim {𝒩}_{n}\left(X\beta ,\sigma_{y}^{2}I_{n}\right)$, where $X\sim {𝒩}_{n}\left(0,\Sigma_{X}\right)$, with an autoregressive correlation structure $\Sigma_{X}={\left(\rho_{X}^{\left|i-j\right|}\right)}_{ij}$, with *μ*_0_ = 0.2, *ρ*_*X*_ = 0.5, and *σ*_*y*_ = 1. To measure and compare predictive performance, we compute the test *R*^2^ based on a test set of *n* = 1,000.

**Figure 1:**
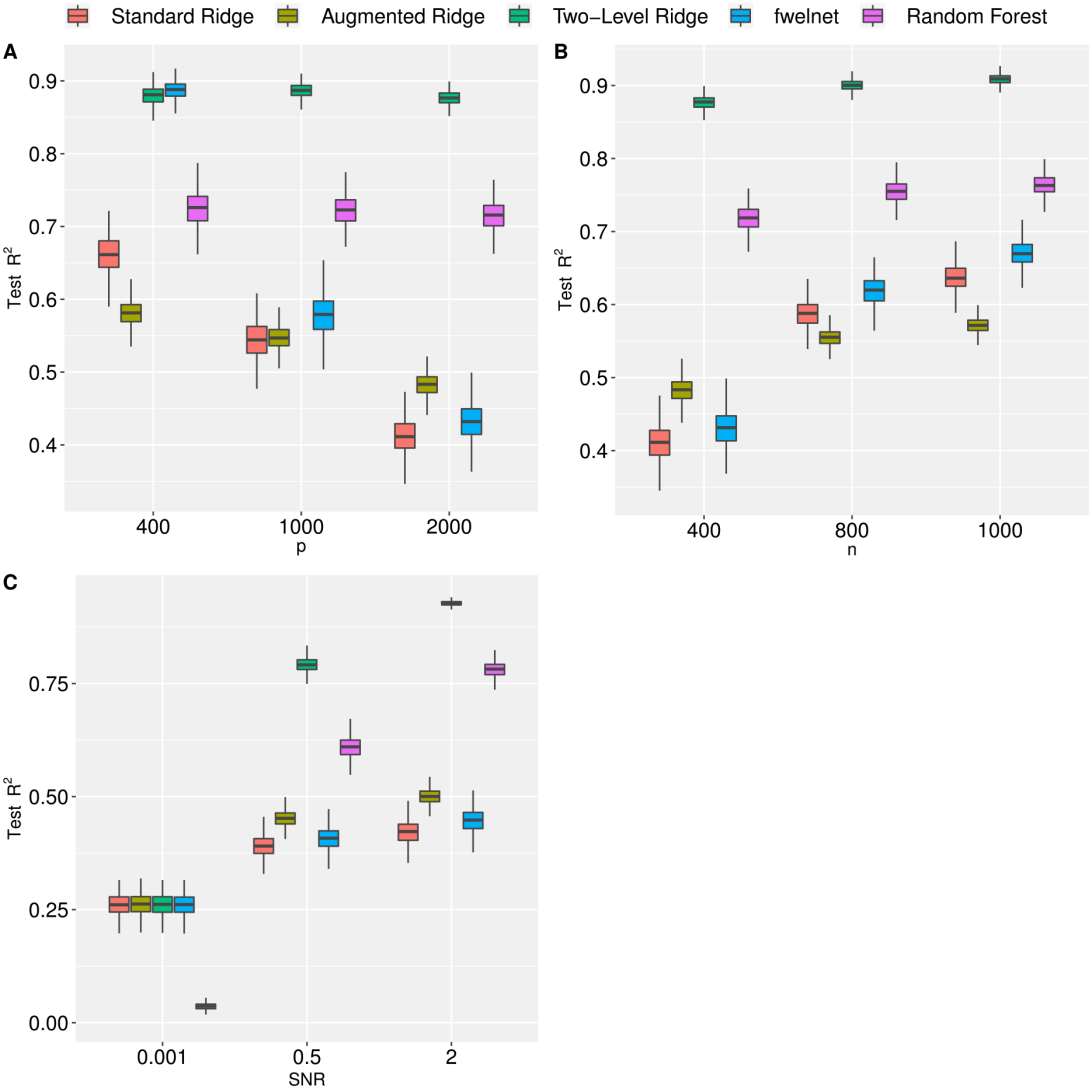
Prediction performance, as measured by test *R*^2^, of standard, augmented, and two-level ridge regression, feature-weighted elastic net (ridge), and random forest by number of features (Panel A), sample size (Panel B), and signal-to-noise ratio (Panel C). In Panel A we fix *n* = 400 and *SNR* = 1. In Panel B we fix *p* = 2,000 and *SNR* = 1. In Panel C we fix *p* = 2,000 and *n* = 400. Results are averaged over 500 Monte Carlo replications. (See Section 3.1 for more information).

In general, we see that two-level ridge regression has better prediction performance when compared to its competitors (Figure 1). As expected, all methods suffer in performance as the number of features increases (Panel A) and improve when the sample size increases (Panel B). In the “small data” scenario (*n* = 1000, *p* = 400, *q* = 6), we observe that fwelnet performs fairly well. However, its performance is comparable to the standard LASSO across several scenarios. This is unsurprising since the outcome is generated assuming that the meta features affect the mean of the subject-level features, not the variance. In both Panels A and B, we set the meta features to be moderately informative (*SNR*_***γ***_ = 1). We evaluate the impact of the informativeness of the meta features by comparing the three methods across a range of *SNR*_***γ***_ (Panel C). With the exception of the random forest algorithm, we see that two-level ridge regression performs similarly to the standard and augmented ridge regression and to fwelnet when the meta features are virtually uninformative (*SNR*_***γ***_ = 0.001) and drastically outperforms them as informativeness increases. We also notice a substantial improvement in the prediction performance of the random forest algorithm as informativeness increases.

### 3.2 Continuous *Z*

Next we simulated data where the meta features are continuous, by drawing *Z* from a multivariate normal density. We let ***γ*** = 0.01 * (**1**_50_, **0**_25_, **3**_25_, **1**_25_, **0**_*q*−150_) and generate *Z*_*p*×*q*_ ~ 𝒩_*q*_(**0**, Σ_*Z*_), where $\Sigma_{Z}={\left(\rho_{Z}^{\left|i-j\right|}\right)}_{ij}$. Similar to Section 3.1, we then simulate $\beta \sim {𝒩}_{p}\left(Z\gamma ,\sigma_{\beta }^{2}I_{p}\right)$ and **y**|*X*, $\beta \sim {𝒩}_{n}\left(\mu_{0}+X\beta ,\sigma_{\text{y}}^{2}I_{n}\right)$, where *X* ~ 𝒩_*p*_(**0**, Σ_*X*_). We fix *μ*_0_ = 0.5, *ρ*_*X*_ = 0.5, *ρ*_*Z*_ = 0, and *σ*_*y*_ = 1. We compare the performance of all five methods across different values of *n*, *p*, *q* and *SNR*_***γ***_.

**Figure 2:**
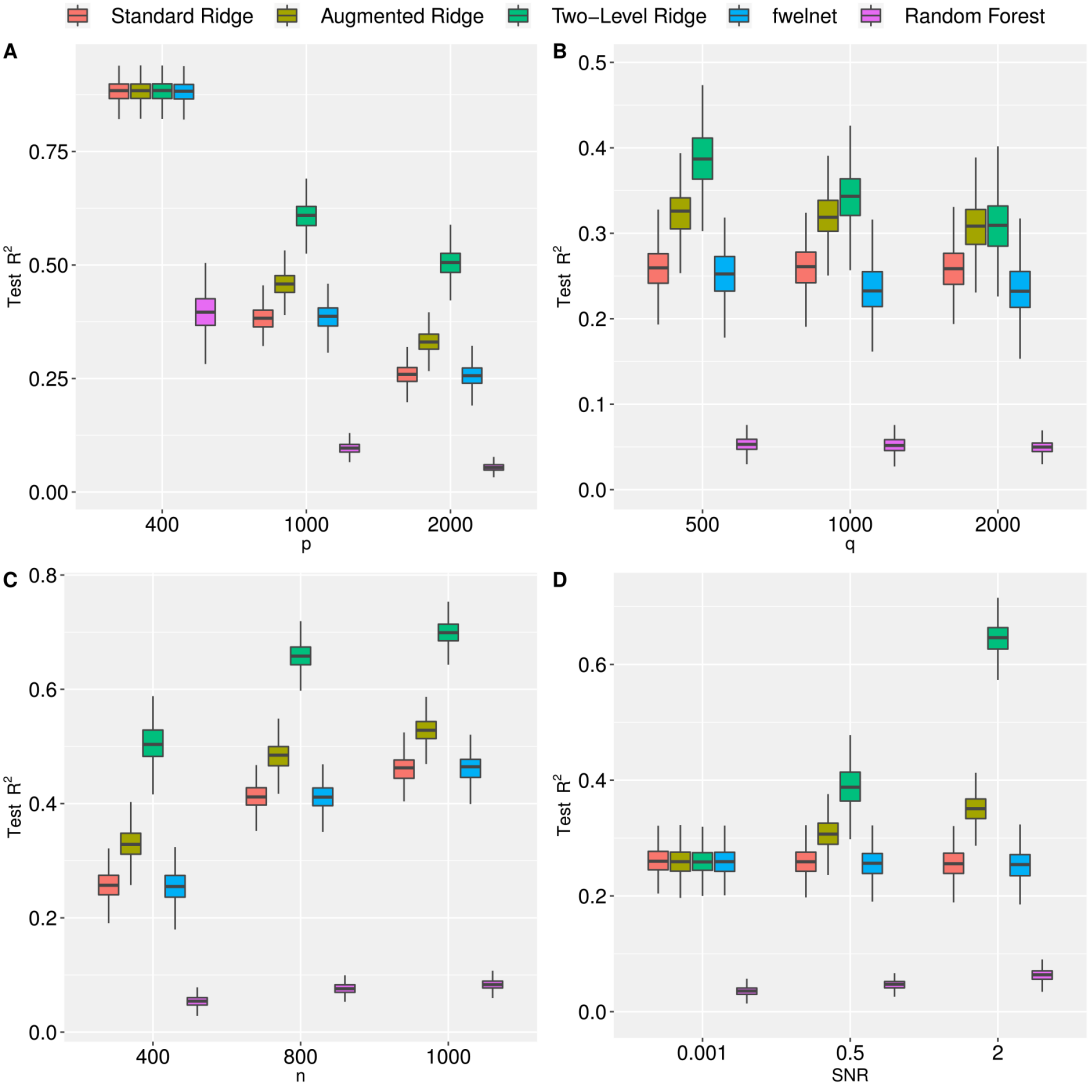
Prediction performance, as measured by test *R*^2^, of standard, augmented, and two-level ridge regression, feature-weighted elastic net (ridge), and random forest by number of features (Panel A), number of meta features (Panel B), sample size (Panel C), and signal-to-noise ratio (Panel D). In Panel A we fix *n* = 400, *q* = 150 and *SNR* = 1. In Panel B we fix *p* = 2,000, *n* = 400, and *SNR* = 1. In Panel C we fix *p* = 2,000, *SNR* = 1 and *q* = 150. In Panel D we fix *p* = 2,000, *q* = 150, and *n* = 400. Results are averaged over 500 Monte Carlo replications. (See Section 3.2 for more information).

Similar to Section 3.1, we consistently see a gain in prediction performance with the two-level ridge regression when compared to its competitors (Figure 2). When the feature dimension *p* increases, there is a degradation in prediction performance across all methods; however, incorporating the meta features in a hierarchical framework outperforms both the standard and augmented ridge methods. The trend was also consistent across varied *σ*_*y*_, *ρ*_*X*_ and *ρ*_*Z*_ (see Figure S1 in the Online Supplementary Materials).

In addition, we also vary the number of meta features in the model (Figure 2 Panel B). Note that as the number of meta features increases, the predictive performance of two-level ridge regression decreases while the performance of standard and augmented ridge regression remain unchanged. The degradation in prediction performance for the two-level ridge regression is expected since we are only increasing the number of noise variables in *Z*. Surprisingly, the random forest algorithm performs poorly in all scenarios.

### 3.3 Binary Outcomes

**Figure 3:**
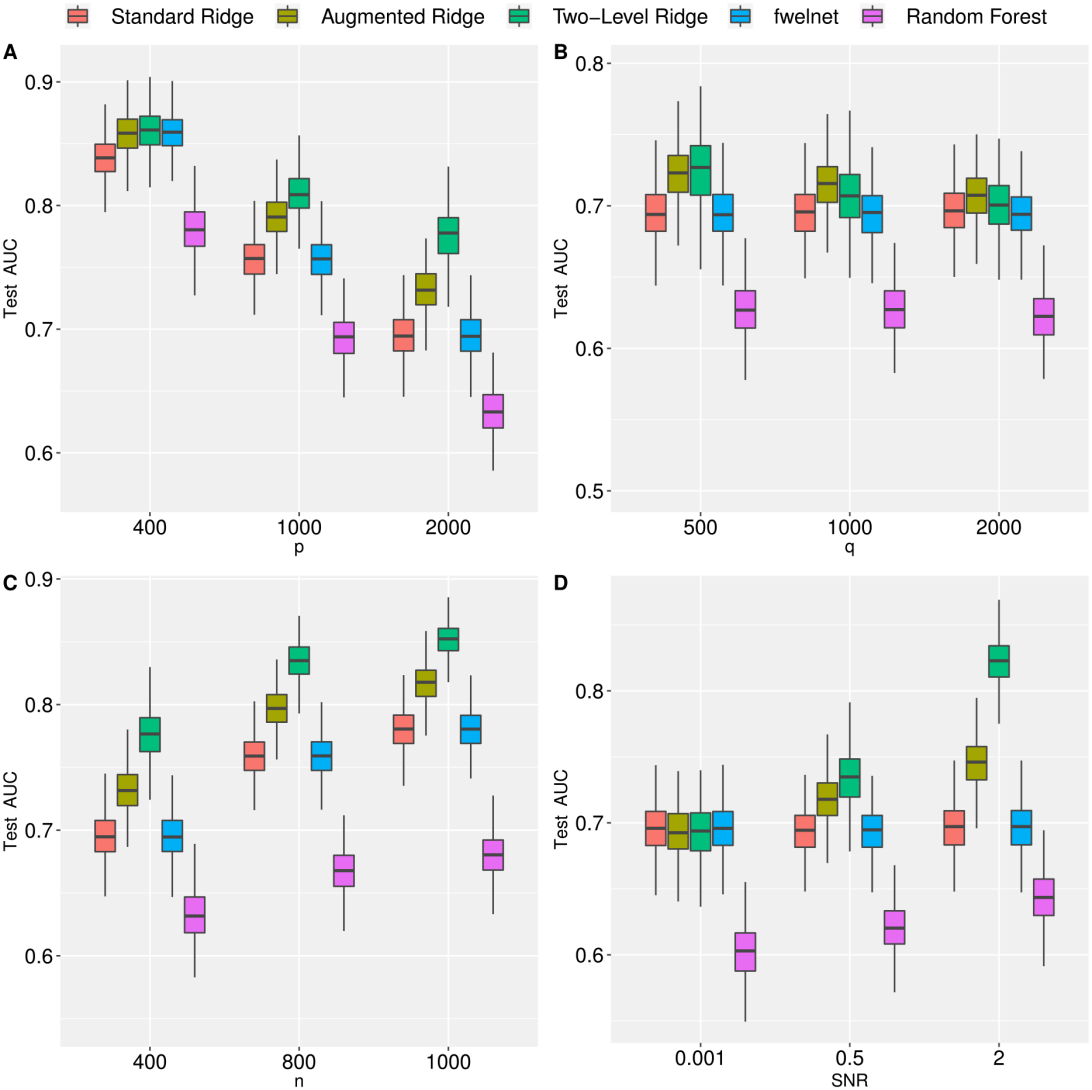
Prediction performance, as measured by test AUC, of standard, augmented, and two-level ridge regression by number of features (Panel A), number of meta features (Panel B), sample size (Panel C), and signal-to-noise ratio (Panel D). In Panel A we fix *n* = 400, *q* = 150 and *SNR* = 1. In Panel B we fix *p* = 2,000, *n* = 400, and *SNR* = 1. In Panel C we fix *p* = 2,000, *SNR* = 1 and *q* = 150. In Panel D we fix *p* = 2,000, *q* = 150, and *n* = 400. Results are averaged over 500 Monte Carlo replications. (See Section 3.3 for more information).

To illustrate two-level ridge regression in a GLM framework, we also compared the performance of all methods under a binary outcome by extending the hierarchical model to logistic regression. The data generating process is similar to Section 3.2 however **y**|*X*, ***β*** ~ *Bernoulli*{*π*(*μ*_0_ + *X****β***)}, where *π*(·) = exp(·)/{1 + exp(·)}. Again, we fixed *μ*_0_ = 0.5, *ρ*_*X*_ = 0.5, and *ρ*_*Z*_ = 0. The true predictive performance was determined as the area under the curve (AUC) for the test set of 1,000 observations. The results are similar to those observed in the continuous case (Figure 3).

## 4 Real Data Applications

### 4.1 Epigenetic Clock

Several studies have demonstrated that DNA methylation levels have strong effects on aging (see e.g., Berdyshev et al., 1967; Rakyan et al., 2010; Teschendorff et al., 2010; Koch and Wagner, 2011; Horvath et al., 2012; Bell et al., 2012). Using DNA methylation levels, epigenetic clocks (see e.g., Hannum et al., 2013; Horvath, 2013) attempt to accurately predict chronological age, with the goal of identifying molecular biomarkers of aging that can be used to study age acceleration and the relationship of methylation and disease (see e.g., Horvath, 2013; Horvath et al., 2015; Levine et al., 2015; Horvath et al., 2016; Quach et al., 2017). High-dimensional regularization techniques have been used to develop these tools. We evaluate the prediction performance of all three ridge regression models (standard, augmented, and two level) on a publicly-available dataset consisting of *n* = 656 individuals with methylation measured on the Infinium 450K platform. The size and structure of the data made competing methods inoperable. Both *xrnet* and *glmnet* permit sparse data structures which allowed us to analyze the data and compared to performance of two-level ridge regression to standard and augmented ridge regression.

While the total number of CpG sites available was 473,034; we reduced the dimensionality of the methylation data by only including the top 250,000 most variable probes. Further, we mapped the methylation probes to the closest gene in terms of physical distance. As meta features of interest we generated the indicators for whether a probe maps to a gene. Thus *Z*, our matrix of external information, consists of *q* columns that represent the *q* unique genes (the *j*th column of *Z* codes all probes that map to gene *j* as one and zero otherwise). After reducing the number of genes in the external data, by only considering genes that have at least 10 probes mapped to them, the resulting *Z* consists of 6,766 unique genes with an average of 33 probes per gene. In our analysis, we normalize *Z* by dividing each column by its sum (i.e. number of probes mapping to the corresponding gene). With this standardization the meta-feature estimate, ${\hat{\gamma }}_{j}$ represents the average effect of all probes that map to gene *j* (*j* = 1, …, *q*) on chronological age. Of note is that both the features (methylation probes) and meta features (gene indicators) are high-dimensional.

We generated 50 training (80%) – test (20%) pairs by randomly splitting the 656 observations. For all three models, 10-fold cross validation is used to tune the penalty parameter(s) in each training data set. Similar to the simulation study, we assessed prediction performance using the test *R*^2^ (averaged across all 50 test sets).

**Figure 4:**
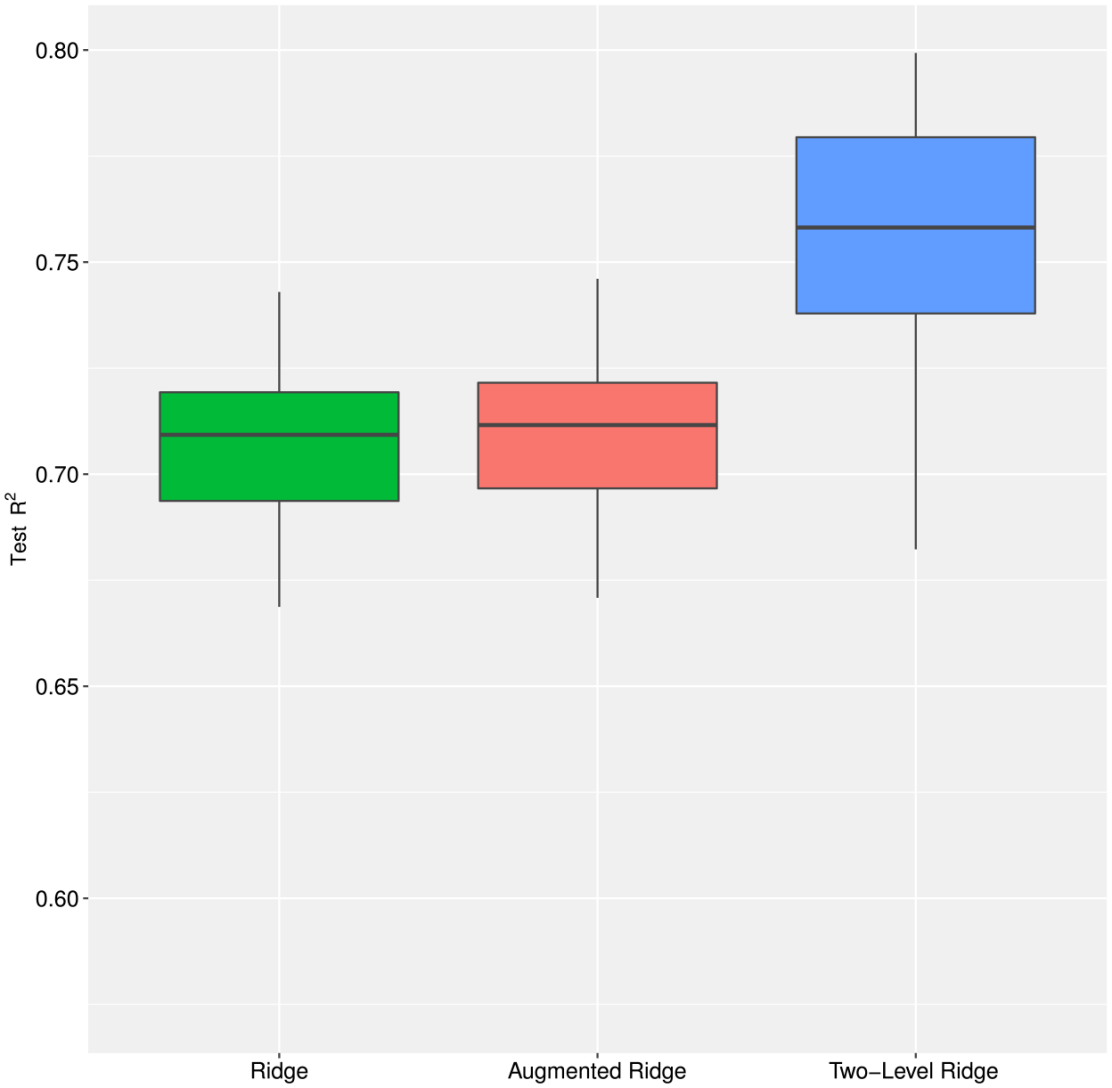
Epigenetic clock: boxplot of test *R*^2^ from 50 training (80%) – test (20%) pairs by randomly splitting the 656 observations. Ten-fold cross validation was used to estimate the tuning parameter(s) for each method. (See Section 4.1 for more information).

The two-level ridge regression significantly improved prediction performance over standard and augmented ridge (Figure 4). The mean test *R*^2^ for standard, augmented, and two-level ridge regression were 0.71, 0.71 and 0.75, respectively, representing a 5.6% improvement in prediction performance when modeling both the methylation probes and their gene groupings hierarchically. By contrast, augmenting the original design matrix by *XZ*, i.e. by the linear combinations of the meta features according to *Z*, did not improve prediction. Our analysis shows that hierarchical regularization, by adequately leveraging external information (i.e., groupings based on genes), can lead to improved performance in predicting chronological age compared to standard approaches for regularization.

### 4.2 Breast Cancer Mortality

We applied the proposed method on a data set of breast cancer tumors from the Molecular Taxonomy of Breast Cancer International Consortium (METABRIC) study available from the European Genome-Phenome Archive (https://ega-archive.org/studies/EGAS00000000083) (Curtis et al., 2012). The data includes cDNA microarray profiling of close to two thousand breast cancer tumor specimens processed on the Illumina HT-12 v3 platform. The METABRIC study was used in an open-source competition (DREAM Breast Cancer Prognosis Challenge) to improve prediction of survival based on clinical characteristics, gene expression levels, and copy number variation. The primary tumors were originally divided into a discovery set of 997 samples and a validation set of 995 samples. In our analysis, we used the discovery set as the training set to fit the model and the validation set as the test set to evaluate the model performance in prediction. The METABRIC dataset also contains the patients’ long-term clinical outcomes and pathological variables (e.g., age at diagnosis, number of positive lymph nodes). Due to significant heterogeneity in expression between ER+/HER2−, ER−, and HER2+ tumors, we restrict our analysis to the subset of patients who were ER+ and HER2−. Furthermore, we dichotomized the patients’ survival time at 5 years and used this binary variable which indicates the 5-year survival of breast cancer as the outcome to predict. The sample sizes, after subsetting to ER+/HER2− patients not censored within 5 years, for the training and test datasets were 594 and 563, respectively and had a mortality (event) rate of 27% and 24%, respectively.

We use the gene expression data, consisting of 29, 477 probes (after pre-filtering), as our primary features in the analysis. A previous study by Cheng et al. (2013), developed a model made of four gene signatures (CIN, MES, LYM, and FGD3-SUSD3), referred to as “attractor metagenes”, that captured molecular events known to be associated with clinical outcomes in many cancers. We generated four meta features by grouping probes that are in the same metagene. In the resulting 29, 477 × 4 matrix, the *j*th column codes all probes that are part of the *j*th metagene as one and zero otherwise. The CIN, MES, LYM, and FGD-SUSD3 metagenes each consist of 61, 70, 69, and 2 genes, respectively. We normalized each column of the meta feature matrix by the number of probes so that each column summed to one.

In addition to comparing two-level ridge regression to both standard and augmented ridge regression, we also implemented the following competing methods: xtune (Zeng et al., 2020), feature-weighted elastic net (fwelnet, Tay et al., 2021) and random forest (Breiman, 2001). The tuning parameter(s) for the five regularized models (two-level ridge, standard ridge, augmented ridge, xtune, fwelnet) were tuned using 10-fold cross validation. For comparison purposes we set the elastic net tuning parameter to 0 for fwelnet, which corresponds to a ridge penalty. A stratification scheme was used to generate the folds due to the class imbalance of cases and controls. Similar to our methylation example, the two-level ridge regression improves class prediction over its competitors (Table 1).

## 5 Discussion

In this paper, we proposed a two-level hierarchical ridge regression model that can directly incorporate meta features into the estimation. We show that the two-level ridge regression can be reformulated into a single-level ridge regression with two tuning parameters, enabling an efficient model coordinate descent fitting algorithm that can handle large numbers of features and meta-features. We provide closed-form solutions under simple scenarios to gain intuition on how the incorporation of meta features impact the estimation of the regression coefficients by borrowing information.

Our simulation results demonstrate that, in general, two-level ridge regression outperforms its competitors when relevant meta features are available. Importantly, in the presence of non-informative meta features, two-level ridge regression has comparable to only slightly worse performance compared to standard ridge regression without meta features. Thus, there is essentially “no cost”, in terms of prediction performance, when incorporating a set of meta features a researcher deems relevant into the model building process. We also illustrate the advantage of our proposed model in two real data applications where we observe improved prediction performance for both continuous and binary outcomes.

We envision several future paths to further improve two-level regularization. First, our current method focuses on incorporating an *ℓ*_2_ penalty for both the subject-level features and meta features. In general, *ℓ*_2_ regularization has been criticized for not being able to perform variable selection (i.e., identifying important predictor variables that are associated with the response of interest), since the ridge penalty shrinks the regression coefficient estimate toward zero, but not exactly to zero. We are currently investigating ways to allow for more general penalties (e.g., LASSO, elastic net, etc.) for both subject-level and meta-feature regularization to allow for variable selection. Second, our real data application focused on five-year mortality as the outcome of interest. While this was done to illustrate the performance of two-level ridge regression for binary outcomes, it would be preferred to model the survival time directly. The Cox (1972) model is a well-appreciated approach to model feature effects on survival (through the conditional hazard function). We are currently developing the two-level regression with a range of penalties, including lasso and elastic net in addition to ridge, as well as a two-level regularized Cox model, which involves replacing the log-likelihood in (9) with the Cox (1975) log-partial likelihood. We expect the implementations of these methods within the two-level regularization framework to provide a wide range of analytical options for integrating prior information into high-dimensional genomic studies.

## Supplementary Material

Supplementary material

## Figures and Tables

**Table 1:** METABRIC study: comparison of the Area under the Curve from a test set (test AUC) of *n* = 563 between standard, augmented, and two-level ridge regression. Model estimation was performed on a training set of *n* = 594. Ten-fold cross validation was used to estimate the tuning parameter(s) for each method. (See Section 4.2 for more information).

Method	Test AUC
Two-Level Ridge	0.69
Ridge	0.67
Aug. Ridge	0.67
fwelnet	0.67
Random Forest	0.67
xtune	0.64
