# Supplementary material for: Hierarchical Ridge Regression for Incorporating Prior Information in Genomic Studies
Source: J Data Sci. Author manuscript; Available in PMC 2023 Jan 1. (PMC9581069; doi:10.6339/21-jds1030)
Supplement: Supplementary material [file NIHMS1831083-supplement-Supplementary_material.zip › JDS2107-007/supplementary.pdf]

Supplementary Files for "Hierarchical Ridge Regression for Incorporating Prior Information in Genomic Studies"

Eric S. Kawaguchi, Sisi Li, Garrett M. Weaver, Juan Pablo Lewinger

S1 Hierarchical Formulation

The ridge regression estimator coincides with the Bayesian MAP (Mode A Posteriori) estimator when the prior for $\boldsymbol{\beta}|\sigma^2 \sim \mathcal{N}(\mathbf{0}, \sigma^2\tau^{-1}I_p)$, where I_p is a $p \times p$ identity matrix and $\tau > 0$. For fixed $\tau_1 > 0$ and $\tau_2 > 0$, we can formulate the two-level ridge regression model similarly with

$$\begin{aligned} \mathbf{y}|X, Z, \boldsymbol{\beta}, \boldsymbol{\gamma}, \sigma^2 &\sim \mathcal{N}_n(X\boldsymbol{\beta}, \sigma^2) \\ \boldsymbol{\beta}|\boldsymbol{\gamma}, \sigma^2 &\sim \mathcal{N}_p(Z\boldsymbol{\gamma}, \sigma^2\tau_1^{-1}I_p) \\ \boldsymbol{\gamma}|\sigma^2 &\sim \mathcal{N}_q(\mathbf{0}, \sigma^2\tau_2^{-1}I_q) \\ \sigma^2 &\sim \pi(\sigma^2), \end{aligned}$$

for some scale-invariant prior $\pi(\sigma^2)$.

S2 Derivations

S2.1 Closed-Form Solution Under Orthogonal X and Z

Assume that both X and Z are orthogonal. Then

$$(\tilde{X}^T\tilde{X} + \Lambda) = \begin{bmatrix} I_p + \Lambda_1 & Z \\ Z^T & I_q + \Lambda_2 \end{bmatrix} = \begin{bmatrix} (1+\lambda_1)I_p & Z \\ Z^T & (1+\lambda_2)I_q \end{bmatrix}.$$

Now

$$(\tilde{X}^T\tilde{X} + \Lambda)^{-1} = \begin{bmatrix} \frac{1}{1+\lambda_1}I_p + \frac{1}{\{(1+\lambda_1)(1+\lambda_2)-1\}(1+\lambda_1)}ZZ^T & -\frac{1}{(1+\lambda_1)(1+\lambda_2)-1}Z \\ -\frac{1}{(1+\lambda_1)(1+\lambda_2)-1}Z^T & \frac{1+\lambda_1}{(1+\lambda_1)(1+\lambda_2)-1}I_q \end{bmatrix}.$$

Therefore

$$\begin{aligned} \hat{\boldsymbol{\theta}} &= (\tilde{X}^T\tilde{X} + \Lambda)^{-1}\tilde{X}^T\mathbf{y} \\ &= \begin{bmatrix} \frac{1}{1+\lambda_1}I_p + \frac{1}{\{(1+\lambda_1)(1+\lambda_2)-1\}(1+\lambda_1)}ZZ^T & -\frac{1}{(1+\lambda_1)(1+\lambda_2)-1}Z \\ -\frac{1}{(1+\lambda_1)(1+\lambda_2)-1}Z^T & \frac{1+\lambda_1}{(1+\lambda_1)(1+\lambda_2)-1}I_q \end{bmatrix} \begin{pmatrix} X^T\mathbf{y} \\ Z^TX^T\mathbf{y} \end{pmatrix} \\ &= \begin{pmatrix} \frac{1}{1+\lambda_1}X^T\mathbf{y} + \frac{1}{\{(1+\lambda_1)(1+\lambda_2)-1\}(1+\lambda_1)}ZZ^TX^T\mathbf{y} - \frac{1}{(1+\lambda_1)(1+\lambda_2)-1}ZZ^TX^T\mathbf{y} \\ -\frac{1}{(1+\lambda_1)(1+\lambda_2)-1}Z^TX^T\mathbf{y} + \frac{1+\lambda_1}{(1+\lambda_1)(1+\lambda_2)-1}Z^TX^T\mathbf{y} \end{pmatrix} \\ &= \begin{pmatrix} \hat{\boldsymbol{\beta}}_{ridge} + \frac{1}{(1+\lambda_1)(1+\lambda_2)-1}ZZ^T\hat{\boldsymbol{\beta}}_{ridge} - \frac{(1+\lambda_1)}{(1+\lambda_1)(1+\lambda_2)-1}ZZ^T\hat{\boldsymbol{\beta}}_{ridge} \\ -\frac{(1+\lambda_1)}{(1+\lambda_1)(1+\lambda_2)-1}Z^T\hat{\boldsymbol{\beta}}_{ridge} + \frac{(1+\lambda_1)^2}{(1+\lambda_1)(1+\lambda_2)-1}Z^T\hat{\boldsymbol{\beta}}_{ridge} \end{pmatrix} \end{aligned}$$

Now since $\boldsymbol{\phi} = \boldsymbol{\beta} - Z\boldsymbol{\gamma}$,

$$
\begin{aligned}
\hat{\boldsymbol{\beta}} = \hat{\boldsymbol{\phi}} + Z\hat{\boldsymbol{\gamma}} = &\ \hat{\boldsymbol{\beta}}_{ridge} + \tfrac{1-2(1+\lambda_1)+(1+\lambda_1)^2}{(1+\lambda_1)(1+\lambda_2)-1} ZZ^T \hat{\boldsymbol{\beta}}_{ridge} \\
&= \left(I_p + \frac{\lambda_1^2}{(1+\lambda_1)(1+\lambda_2)-1} ZZ^T \right) \hat{\boldsymbol{\beta}}_{ridge} \\
&= \left(I_p + \frac{\lambda_1^2}{\lambda_1\lambda_2 + \lambda_1 + \lambda_2} ZZ^T \right) \hat{\boldsymbol{\beta}}_{ridge}
\end{aligned}
$$

S3 Cyclic Coordinate Descent Algorithm for Two-Level Ridge Regression

Recall $\boldsymbol{\theta} = (\boldsymbol{\phi}, \boldsymbol{\gamma})$ and $\tilde{X} = [X, XZ]$. Optimization via cyclic coordinate descent is straightforward. We start by setting all $p+q$ variables to some initial value (e.g., $\boldsymbol{\theta}^{(0)} = \mathbf{0}$, where the superscript identifies the iteration of the algorithm. At the $(m+1)$-th iteration, a series of one-dimensional updates are performed until the algorithm cycles through all the variables, returning $\hat{\boldsymbol{\theta}}^{(m+1)}$. The cycling process is repeated until some convergence criterion $l(\hat{\boldsymbol{\theta}}^{(m)}, \hat{\boldsymbol{\theta}}^{(m+1)}) < \delta$ for some $\delta > 0$ is met (e.g., $l(a,b) = ||a-b||_2^2$).

S3.1 Ordinary Least Squares

For the ordinary least squares model, one can show that the one-dimensional update for the j-th variable at the $(m+1)$-th iteration is

$$\hat{\theta}_j^{(m+1)} \leftarrow \frac{\tilde{\mathbf{x}}_j^T \left(\mathbf{y} - \tilde{X}_{-j} \hat{\boldsymbol{\theta}}_{-j}^{(m)} \right)}{\tilde{\mathbf{x}}_j^T \tilde{\mathbf{x}}_j + \lambda_j}, \tag{1}$$

where $\tilde{\mathbf{x}}_j$ is the j-th column of $\tilde{X}$, $\tilde{X}_{-j}$ is $\tilde{X}$ without the j-th column, $\hat{\boldsymbol{\theta}}_{-j}^{(m)}$ is $\hat{\boldsymbol{\theta}}^{(m)}$ without the j-th element, and $\lambda_j = \lambda_1$ if $j \in \{1, \ldots, p\}$ and equal to λ_2 if $j \in \{p+1, \ldots, p+q\}$.

S3.2 Generalized linear models

Letting $\nabla l(\boldsymbol{\theta}) = \partial l(\boldsymbol{\theta}) / \partial \boldsymbol{\theta} = \tilde{X}^T \mathbf{u}$ and $\nabla^2 l(\boldsymbol{\theta}) = \partial^2 l(\boldsymbol{\theta}) / \partial \boldsymbol{\theta} \partial \boldsymbol{\theta}^T = \tilde{X}^T W \tilde{X}$, we approximate the log-likelihood based on a Taylor series expansion about the current iteration $\boldsymbol{\theta}^{(m)}$:

$$l(\boldsymbol{\theta}) \approx \frac{1}{2} (\tilde{\mathbf{y}} - \tilde{X}\boldsymbol{\theta})^T W (\tilde{\mathbf{y}} - \tilde{X}\boldsymbol{\theta}),$$

where $\tilde{\mathbf{y}}$ is the working response vector $\tilde{\mathbf{y}} = X\boldsymbol{\theta}^{(m)} + W^{-1}\mathbf{u}$. Note here that $\mathbf{u}$, W, and $\tilde{\mathbf{y}}$ are dependent on $\boldsymbol{\theta}^{(m)}$. We can use cyclic coordinate descent to minimize (11). For the two-level ridge regression for GLMs, the one-dimensional update for the jth variable at the $(m+1)$-th iteration is

$$\hat{\theta}_j^{(m+1)} \leftarrow \frac{r_j}{v_j + \lambda_j}, \tag{2}$$

where v_j is the jth diagonal element of $V = \tilde{X}^T W \tilde{X}$ and r_j is the jth element of $\mathbf{r} = \tilde{X}^T W \mathbf{u} + V \boldsymbol{\theta}^{(m)}$.

S4 Additional Figures

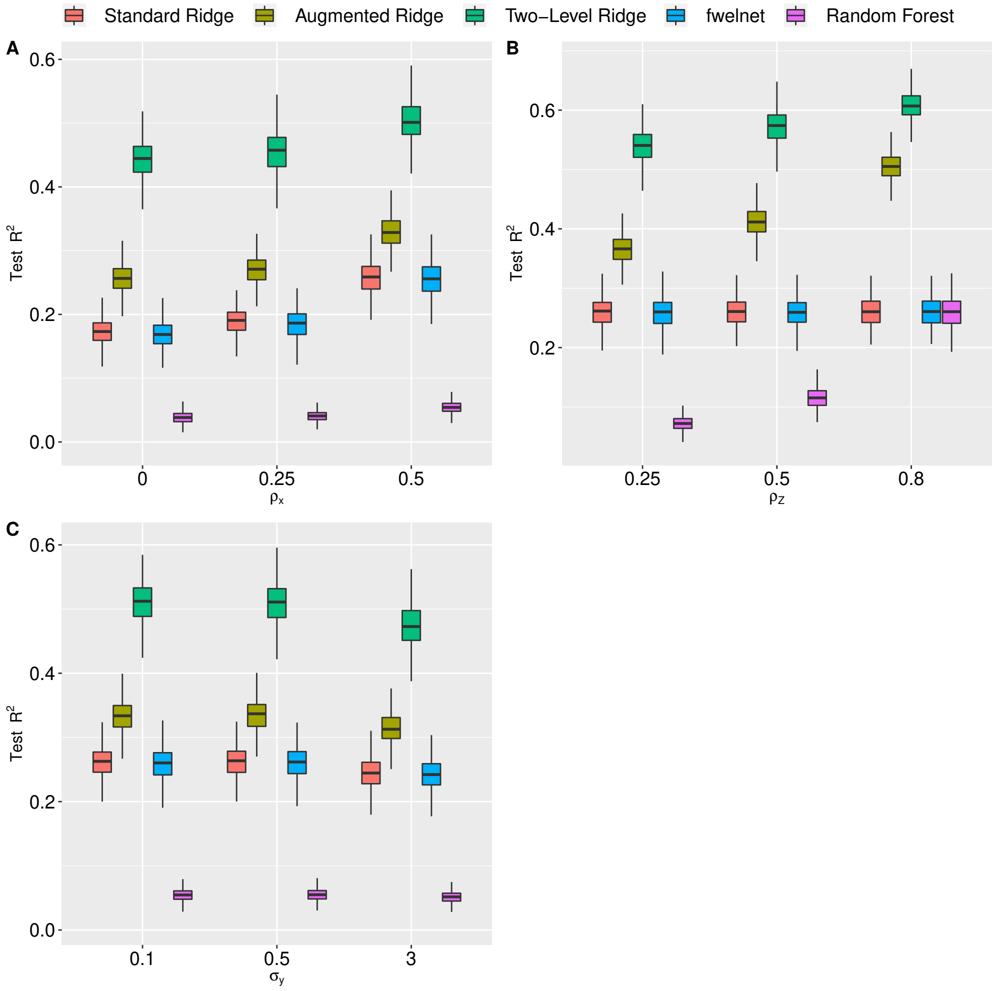

Figure S1: Prediction performance, as measured by test R^2, of standard, augmented, and two-level ridge regression by ρ_X (Panel A), ρ_Z (Panel B), and σ_y (Panel C). In all panels we fix $n = 400$, $p = 2,000$ and $q = 150$. In Panel A we fix $\rho_Z = 0$ and $\sigma_y = 1$. In Panel B we fix $\rho_X = 0.5$ and $\sigma_y = 1$. In Panel C we fix $\rho_X = 0.5$ and $\rho_Z = 0$. Results are averaged over 500 Monte Carlo replications. (See Section 3.2)
